# Ventricular Arrhythmias in Hypertrophic Cardiomyopathy- Can We Ever Predict Them?

**Published:** 2010-03-05

**Authors:** Narayanan Namboodiri, Johnson Francis

**Affiliations:** 1 Sree Chitra Tirunal Institute for Medical Sciences and Technology, Trivandrum, Kerala, India; 2 Malabar Institute of Medical Sciences, Calicut, Kerala, India

**Keywords:** hypertrophic cardiomyopathy, ventricular arrhythmias

Hypertrophic cardiomyopathy (HCM) is characterized by gross cardiac and myocyte hypertrophy, myocyte disarray, and interstitial fibrosis. This condition is relatively common, with a prevalence of about 1:500 in the general population. Most patients with HCM are either asymptomatic or have only minimal symptoms. In general, HCM is a relatively benign disease with an annual mortality rate of slightly less than 1% in unselected HCM populations [1,2]. However, sudden cardiac death (SCD) may be the first manifestation of the disease. Approximately 60% to 70% of all patients with HCM die suddenly [3], and the fatal event is generally assumed, though not proven so far, due to ventricular arrhythmias.

The high arrhythmic propensity in HCM is contributed by a combination of many primary substrate abnormalities like hypertrophy, myocardial fibre disarray, interstitial fibrosis etc, and possible secondary triggers like ischemia, physical exercise, and excessive sympathetic stimulation.  Marked heterogeneity in substrate and triggers would substantially reduce the predictive accuracy of any risk stratification model as known to occur in patients with many other cardiac arrhythmic conditions. To compound this, heterogeneity in the studied population, low event rate, effect of drug therapy etc would also limit the application of any predictive model.

However, a few revelations in the last decade have contributed to the better understanding of the fatal arrhythmia risk. Nonsustained ventricular tachycardia (NSVT) detected on Holter monitoring in younger (less than 30 years) patients have been identified to be associated with higher mortality rate [4]. Unlike their older counterparts where myocyte loss and fibrosis contribute incrementally to arrhythmogenicity, the higher mortality risk in younger patients probably reflects a more potent arrhythmogenic substrate caused by myocyte disarray, myocardial ischemia, and abnormal autonomic function. No doubt, the association between SCD and NSVT in young patients is striking; however, the majority of SCDs, even in young patients, occurred in patients without NSVT. This clearly shows that Holter monitoring identifies only a subset of subjects at higher risk. Clearly we need to rule out other contributing risk factors before reassuring an individual patient based on this non-invasive modality only.

Is there any role for 12-lead electrocardiogram to predict arrhythmia risk in them? In a cohort of patients with HCM selected because of their high risk for SCD, none of the studied electrocardiographic features (markedly increased voltages, QRS duration, left or rightward QRS axis, abnormal Q waves, and QTc or QT dispersion) did not predict subsequent appropriate implantable defibrillator intervention for ventricular tachyarrhythmias and was not useful in risk stratification for SCD [5]. Similarly, T wave alternans was also not useful to predict SCD in patients with HCM [6].

Another possible predictor could be exercise-induced arrhythmias. Though occurrence of ventricular arrhythmias during exercise is rare in HCM, its presence has long been identified to be associated with an increased SCD risk. In a recent study, the presence exercise-induced nonsustained VT or ventricular fibrillation was associated with a 3.73-fold increase in the risk of SCD or hemodynamically compromising sustained VT during follow-up [7].

Of late, a few studies with cardiac magnetic resonance (CMR) imaging in HCM also have shown some promising insights into risk stratification [8-12]. Delayed enhancement in CMR in HCM correlates with the histological finding of fibrosis and thus represents a likely substrate for ventricular tachyarrhythmias. In asymptomatic or mildly symptomatic HCM patients this finding in CMR had a significantly increased frequency of ventricular tachyarrhythmias on Holter monitoring compared with those without it. However, larger studies are required before establishing the role this non-invasive tool in the risk stratification in HCM.

Is there a role of genotype in deciding risk of SCD in these patients? Since the discovery of first causal gene and mutation for HCM in 1990, more than a dozen sarcomeric genes have been implicated in this disease [13]. Of these, certain mutations are considered high risk - for example, most mutations in TNNT2, R719Q and R403Q in MYH7, and double-causal mutations. However, a myriad of genetic (modifier genes, microRNAs, post-translational modifications of proteins, epigenetic factors etc) and nongenetic factors interplay to result in the complex phenotype in HCM. So a predictive model to assess the global risk of SCD in HCM based on genotype alone is still far from reality.

In conclusion, despite being half a century down the initial detailed clinical description of the entity, the predictors of fatal arrhythmias in this disease still remain largely elusive. However, in future, more advanced research into the causal genes and genotype-phenotype correlation, and larger natural history studies may likely enable us to predict the arrhythmia risk in HCM in a better way.
